# Health providers’ perspectives on effects of the COVID-19 pandemic and anti-epidemic measures on maternal health services in Nairobi, Kenya: a qualitative study

**DOI:** 10.1186/s12884-025-07500-8

**Published:** 2025-04-11

**Authors:** Cynthia Khamala Wangamati, Paul Wenzel Geissler, Erick Otieno Nyambedha, Ruth Jane Prince

**Affiliations:** 1 Department of Community Medicine and Global Health, Institute of Health and Society, University of Oslo, Postboks 1130 Blindern, Oslo, 0318 Norway; 2 Department of Social Anthropology, University of Oslo, Oslo, Norway; 3 Department of Sociology and Anthropology, Maseno University, Maseno, Kenya

**Keywords:** COVID-19, Maternal health, Pregnant women and mothers, Health providers, Kenya

## Abstract

**Background:**

The first case of COVID-19 in Kenya was confirmed in March 2020; the Kenyan government swiftly introduced measures to curb transmission, some of which negatively impacted maternal health services. Most research on the effects of COVID-19 on maternal health is from the perspectives of pregnant women and mothers. Our study explores health providers’ perspectives on the effect of COVID-19 on maternal health services in Nairobi, Kenya.

**Methods:**

From February to May 2023, we conducted key informant interviews with 39 health providers in Nairobi County, Kenya. Study participants included medical doctors/officers, clinical officers, nurses, and community health assistants. Thematic inductive and deductive analysis were used to analyze the data; coding was done using NVIVO.

**Results:**

The interviewed health providers confirmed that COVID-19 prevention and infection control measures had negatively affected maternal health services. The measures led to the temporary closure of health facilities because there was a shortage of health providers due to their deployment in isolation centers and quarantine, and some contracted COVID-19, restricted access to maternal health services, and delayed service delivery due to social distancing. Health providers stated that they faced numerous challenges, including fear of and contracting COVID-19, being overworked as they were short-staffed, limited resources, stigma from the community as they were considered infectious, and psychological distress. In addition, health providers said that the Kenyan government addressed some challenges by providing personal protective equipment, prioritizing health providers for vaccination, facilitating their movement, and providing temporary tax relief.

**Conclusion:**

Our findings highlight the negative effect of COVID-19 on maternal health services in Kenya. Future pandemic preparedness should entail proper planning, staffing, training, psychosocial support, and staff motivation through the provision of risk allowance and health insurance. In addition, funds should be set aside for purchasing medical supplies, equipment, and vaccines, building isolation centres, and other contingencies.

**Supplementary Information:**

The online version contains supplementary material available at 10.1186/s12884-025-07500-8.

## Introduction

The World Health Organisation (WHO) estimated the global maternal mortality rate (MMR) in 2020 at 223 maternal deaths per 100.000 live births; sub-Saharan Africa had the highest MMR globally, estimated at 545 maternal deaths per 100,000 live births, accounting for approximately 70% of global maternal deaths in 2020 [1]. In Kenya, MMR is estimated at 530 deaths per 100,000 live births [1]. Globally, there were reports of increased maternal deaths, stillbirth, ruptured ectopic pregnancies, and maternal depression during the COVID-19 pandemic [2]. A rapid systematic review of maternal mortality during the COVID-19 pandemic in low- and middle-income countries yielded seven studies that reported increased levels of maternal mortality, of which four were statistically significant [3].

Studies conducted in sub-Saharan Africa (SSA) indicate that COVID-19 had a negative impact on maternal healthcare services. A mixed-methods study that compared maternal healthcare utilization in six referral hospitals in four SSA countries before and during the COVID-19 pandemic reported that service utilization declined compared with the pre-pandemic year in all except one referral hospital [4]. The study, conducted in Guinea, Nigeria, Tanzania, and Uganda, attributed the decline to fear of being infected with COVID-19 in health facilities, lack of transportation or high cost of transportation, and service closures due to self-isolating health providers. The same study found that during the first wave of COVID-19, health providers were provided with limited knowledge of COVID-19, lacked infection prevention and control training, and experienced difficulties reaching their workplaces [5]. The challenges that persisted beyond the first wave were a shortage of personal protective equipment and a lack of rapid testing for women suspected of COVID-19. A literature review on the impact of COVID-19 on maternal and child health in Africa reported psychological stress in pregnant women and mothers, and reduced antenatal care attendance, childhood vaccination, and facility-based births due to poor health facilities utilization because of contracting COVID-19 fear, receiving care delays, and COVID-19 mitigation measures [6].

Studies on other pandemics have shown a negative impact on maternal health. A systematic review on the effect of Ebola virus disease on maternal health service utilization and perinatal outcomes in West Africa reported a decrease in institutional deliveries and antenatal care visits during the Ebola pandemic compared to the pre-Ebola period [7].

After the first case of COVID-19 was confirmed in March 2020, the Kenyan government rapidly introduced measures to curb transmission. They included the ban on international travel and travel between counties, night curfews, mandatory quarantines in isolation centres, the mandatory wearing of facial masks in public spaces, the closure of schools and institutions of higher learning, and the closure of clubs, restaurants, and non-essential businesses [8]. Some of these measures were modified during the pandemic, and later lifted as the number of COVID-19 infections decreased, and a sizeable Kenyan population were vaccinated against COVID-19. Table 1 shows infection rates and measures taken during the period with high COVID-19 infection rates in Kenya [9–11].

**Table 1:** Timeline of COVID-19 infection and prevention measures in Kenya

Date	Event
28th February 2020	National Emergency Response Committee established.
6th March 2020	COVID-19 treatment and isolation facility at Mbagathi Hospital established.
13th March 2020	The first COVID-19 positive case confirmed
14th March 2020	Activation of the Public Health Emergency Operation Center.
15th March 2020	Travel from other countries restricted; quarantine upon entering country; work from home; no social gatherings
25th March 2020	Nationwide curfew between 19h00–05h00 put in place
28th March 2020	Mandatory quarantine for positive patients and their contacts imposed
3rd April 2020	Government extends the mandatory quarantine period beyond 14 days
6th April 2020	Masks mandatory in public places, containment measures across Nairobi, Kilifi, Kwale and Mombasa restricting travel in and out of the counties.
7th April 2020	Mass testing of all health workers and medical staff
16th April 2020	A 3-month waiver on county governments’ purchases of masks and PPEs and the development of a welfare package for frontline officers, e.g., medical insurance.
25th April 2020	Extension of cessation of movement into and out of Nairobi Metropolitan Area, Mombasa, Kilifi and Kwale counties for 21 days & the national curfew for 21 days. Tax Laws Bill, 2020 with various tax reliefs for citizens & companies.
16th May 2020	Extension of cessation of movement into and out of Nairobi Metropolitan Area, Mombasa, Kilifi and Kwale counties for 21 days
6th July 2020	-Lifting of the intercountry cessation of movement in and out of Nairobi, Mombasa and Mandera effective 7 July 2020; - Extension of nationwide curfew from 21h00 and 04h00 daily for a month
26th August 2020	Nationwide curfew extended for a month
28th September 2020	Nationwide curfew in force throughout the territory of Kenya for a further 60 days between 21h00 to 23h00
12th October 2020	Schools reopen starting with examination classes for students
4th November 2020	Political gatherings and rallies suspended; nationwide curfew extended to 03.01.2021 from 10h00 to 04h00.
January 2021	All schools to reopen
March 2021	Vaccinations started of frontline workers and individuals older than 58years
24th March 2021	Five counties including Nairobi put on lockdown
2 May 2021	Lockdown lifted in the respective counties

Studies on the impact of COVID-19 on maternal health and maternal health services in Kenya report that it negatively impacted services; pregnant women and mothers reported fear of contracting the disease and being isolated after testing positive as a hindrance to seeking maternal health services [12–14]. Consequently, pregnant women delayed seeking antenatal services [15], pregnant women and mothers missed scheduled appointments for ante-and postnatal care services, and there were increased home deliveries and deliveries by traditional birth attendants (TBAs), and increased maternal mortality [13, 14]. Moreover, some pregnant women and mothers experienced economic hardships due to the overall reduction of economic activity that made them unable to afford health services [13, 16]. In addition, health system resources allocated for maternal health programs were diverted to address COVID-19, and some health facilities were temporarily shut down due to inadequate resources and poor preparation of health workers to deal with the pandemic [14].

Most research on the effect of COVID-19 on maternal health services in Kenya is from the perspectives of pregnant women and mothers [12, 17–20]. Few have focused on health providers’ perspectives, challenges, and recommendations for future pandemic preparedness [14]. The aim of our study was to explore health providers’ perspectives on the effect of COVID-19 on maternal health services in Nairobi, Kenya. This study is relevant because it highlights vulnerability, resilience, motivation, and human and financial resource-related factors relevant to informing measures to prepare for future crises [21].

### Methods

#### Study design

This exploratory, descriptive qualitative study employed key informant interviews with health providers to explore and describe their perspectives on the effect of COVID-19 on maternal health services and their work based on their experiences of the pandemic [22]. The research approach was used because it helps researchers to draw out concepts, opinions, and other factors shaping experience and perception [23, 24]. It enabled the authors to understand the real-world context experienced by health providers during the COVID-19 pandemic [25].

### Settings

The study was conducted in Dagoretti North and Dagoretti South sub-counties in Nairobi County, Kenya. According to the 2019 Kenya Population and Housing Census Volume III, Nairobi has an estimated population of 4,396,828; Dagoretti North and South sub-counties have an estimated population of 434,177 [26]. In the initial stages of the pandemic in Kenya, Nairobi County was the epicenter of COVID-19 infections; the study area, which mainly comprised informal settlements, was among areas reported to be COVID-19 hotspots [27]. The study was conducted in four public health facilities, two in Dagoretti North and two in Dagoretti South sub-counties. The health facilities were 2 level 3-Health centres, which offer antenatal, postnatal care and have child welfare clinics (Riruta Health Center and Statehouse Dispensary); one level 3b Health centre that offers antenatal and postnatal care, has a child welfare clinic as well as skilled birth deliveries- not cesarean (Waithaka Health Center); and one level-4 sub-county hospital that offers antenatal, skilled birth deliveries including cesarean deliveries and postnatal services, and has a child welfare clinic (Mutuini sub-District Hospital). These facilities serve mostly the inhabitants of informal settlements in Nairobi.

### Participant recruitment

We used purposeful and snowballing sampling to identify and collect data from health providers working on maternal health between 2019 and 2023 during the COVID-19 pandemic in the selected health facilities [28]. In three health facilities, i.e., Riruta Health Center, Statehouse Dispensary, and Mutuini sub-District Hospital, the facility-in-charges appointed a health provider who recruited the participants on our behalf. This is because they knew which departments the health providers worked in, whether they had been at the facility during the pandemic, and their interview availability (off duty). The facility-in-charge asked us to recruit the health providers independently in one health facility, Waithaka Health Center. There, the first author managed to interview one health provider, who, through snowballing, referred us to other health providers who met our selection criteria. Before the commencement of the interviews, we sought informed written consent and assured the participants that their participation was voluntary and that there were no repercussions for not participating.

### Data collection

Data were collected from February to May 2023. The first author conducted the key informant interviews using semi-structured interview guides (Supplementary file 1). After the interviews, the first author took notes (field notes). The notes contained information about the (i) study title, (ii) the author’s name, (iii) people interviewed, (iv) time, (v) geographical settings (physical environment where the interviews took place), (vi) non-verbal responses of research participants to questions, and (vii) the author’s reflection on the interview process [29]. The audio-recorded interviews took place in private spaces at the workplace of the research participants. We interviewed health providers to the point of saturation, i.e., when we felt that no new information was being obtained from the interviews [30]. The saturation point was reached when additional interviews reproduced information gathered in earlier ones, without adding variation. Since there was no variation, all authors on the research team agreed that we had exhausted our line of inquiry. The interviews were conducted in English (language used at the place of work), lasted between 30 and 90 min, and were transcribed verbatim by an independent transcriber. The themes explored were, among others, the effect of COVID-19 on maternal health services, challenges experienced by health providers during this period, government support, and health providers’ recommendations on emergency preparedness.

### Data analysis

Data were analyzed deductively and inductively; thematic analysis was used to identify, analyze, and report patterns within the data [31].

The iterative thematic deductive and inductive analysis entailed familiarization with the data, generation of initial codes, theme search, theme review, and theme definition and naming [32]. After data collection, transcripts and field notes were read and reread to gain an in-depth understanding of the data [31] and coded using NVIVO 14 by the first author. Initially, the first author conducted the analysis inductively and developed a table of themes and sub-themes from generated codes. In addition, the first author applied the predefined themes and sub-themes developed priori from the interview guide, such as the effect of COVID-19 on maternal health services, challenges, pandemic preparedness, etc., to the inductively generated codes. The themes and sub-themes from the inductive and deductive analysis were transferred from the NVIVO software, put in a table format in an MS Word document with supporting text, and presented to the other three authors. The authors then reviewed and deliberated on each table of themes and subthemes (from inductive and deductive analysis). The authors agreed upon four themes based on the frequency of themes across the tables, coherence, and strength of data supporting each identified theme. The field notes were incorporated to provide context to the quotes in the results section.

### Trustworthiness

The authors ensured trustworthiness by focusing on credibility, transferability, dependability, and confirmability [33]. The first author is a researcher based at the University of Oslo, Norway, but Kenyan with a background in public health. The second and last authors are professors in medical anthropology at the University of Oslo. The third author is a professor in social anthropology at Maseno University in Kenya. Credibility was maintained through interviewing different cadres of health providers in different health facilities. In addition, the first author observed some measures enacted during the COVID-19 pandemic that remained in the health facilities but had been repurposed or were no longer in use. During the interviews, the first author observed that some facilities were using isolation centres (enacted tents), which had been converted for use to host health education sessions with clients, and observed seats with stickers indicating social distancing and numbers to call for ambulatory care on walls in health facilities. To facilitate the transferability of research findings, the authors have described the study context, participants, and data collection and analysis procedures. For the study’s dependability, the authors have shared their interview guides (supplementary file 1) and documented their data collection and analysis procedures for reproducibility. After every interview, member checking was conducted by the first author. She summarised the study findings to ensure she correctly represented the health providers’ perspectives. After the end of the interview, health providers were requested to clarify misunderstandings, add anything that might have been left out by the interviewer, and permit the researcher to use the findings. Some participants [4] permitted only part of the interview to be used after the summary of findings.

### Ethical considerations

The study protocol was approved the Norwegian Agency for Shared Services in Education and Research (SIKT) and Maseno University Ethics Review Committee (MUERC/01063/22). In Kenya, we secured a research permit from the National Commission for Science, Technology & Innovation (NACOSTI/P/23/31485). We approached 46 study participants; however, only 39 participated. Written informed consent was obtained from all participants after explaining the study objectives, possible risks and discomforts, benefits, the confidentiality of information, and data management. Participants were assured of privacy and confidentiality and informed that participation was voluntary and that they could withdraw at any time from the study without any repercussions. The participants were reimbursed KES 1000 (Euro 7) for participation in the study. The ethical considerations were in accordance with the Declaration of Helsinki.

## Results

### Sociodemographic characteristics

We interviewed 39 health providers, namely medical doctors/officers, clinical officers, nurses, and community health assistants, of whom 33 were women and 6 were men. They had worked in maternal health from late 2019 to May 2023. Table 2 shows the participants’ socio-demographic characteristics.

**Table 2:** Sociodemographic characteristics

Profession	Age range	Riruta	Waithaka	Mutuini	Statehouse	Total
Doctors	21–30	0	0	1	0	1
Clinicians	21–30	0	0	1	0	1
	31–40	0	1	1	0	2
Nurses	21–30	0	0	3	0	3
	31–40	3	5	6	2	16
	41–50	0	1	4	2	7
	51–60	3	3	0	0	6
Community Health Assistants	31–40	2	0	0	0	2
	41–50	0	1	0	0	1
	**Total**	8	11	16	4	39

### Themes and sub-themes

The authors agreed on four themes: (i) the effect of COVID-19 on maternal health services, (ii) challenges experienced by health providers, (iii) the government response to challenges experienced, and (iv) pandemic preparedness. Table 3 details our coding process.

**Table 3:** Themes, sub-themes, and codes

Themes	Sub-themes	Codes
1. Effect of COVID-19 on maternal health services	Temporary closure of health facilities; shortage of health providers; limiting numbers of clients to be seen; limiting services offered; delayed service delivery	Health facility closed; referral to other health facilities; health providers taken to COVID-19 quarantine centers; few clients because of social distancing; clients and birth companions told not to come to health facilities; avoiding contact with patients; limiting hospital stays after delivery; no access to long-term family planning services; prioritization of some health services
2. Challenges experienced by health providers	Fear of contracting COVID-19; contracting COVID-19; stigma; overworking; limited resources; psychological stress	Fear of clients; fear of infection; effects of COVID-19 on health; effects of COVID-19 on finances; neighbors restricting contact; being labeled as COVID-19 people; long shifts; feeling overwhelmed; lack of PPEs; lack of ambulatory care; limited access to transport; psychological disturbance; poor mental health
3. Government response to challenges experienced	Training on COVID-19 prevention and infection control; supply of PPEs & vaccination; tax relief; travel pass	Education of health providers, community, and clients; masks; PPEs, vaccines; reduced taxes; travel permit
4. Pandemic preparedness	Awareness creation and training of health providers; financial and human resources; isolation centers; counseling; motivation of health providers	Training; funds set aside; isolation centres, psychological trauma; stress relief; compensation

### Theme 1: effect of COVID-19 on maternal health services

According to the research participants, COVID-19 prevention and infection control measures affected the delivery of maternal health services. They explained how the measures led to the temporary closure of health facilities, a shortage of health providers, and limited health care services to pregnant women and mothers seeking health services. Below we draw upon direct quotes that illustrate many of the statements health workers made during the interviews.

#### Sub-theme 1.1: temporary closure of health facilities

During the early period of COVID-19, some health providers in two health facilities contracted COVID-19. Consequently, the health facilities were closed for about 2 weeks to ensure that health providers were COVID-19-free before they could resume work. This led to patients being referred to other health facilities. According to health providers, the closure of the health facilities made some patients associate the facility with COVID-19 and did not seek health services from the facility when it reopened for fear of contracting the virus. A health provider stated:

*…. there was an outbreak of COVID-19*,* most of our staff had COVID-19*,* and our facility was closed for two weeks. So*,* you see*,* there were no services at that time. So*,* by the time you bring them (clients)…. they also fear because most staff had COVID-19*. (IDI-002-Riruta)

#### Sub-theme 1.2: shortage of health providers

At the peak of COVID-19, health providers were taken to COVID-19 isolation centers to manage patients, creating a shortage of staff providing other services. As a result, health providers working on maternal health reported being overwhelmed, which compromised their ability to provide quality care, especially during deliveries. In addition, when health providers contracted COVID-19, they were supposed to isolate, reducing the number of available health providers. A health provider elaborated:

*… You find that you have like four mothers in labour*,* and you are supposed to be two*,* and you find others are taken to COVID centers. So you find yourself handling 5 mothers*,* and you are just alone*,* and you are supposed to be like two… I have to give the service very fast*,* so I will miss something. I may be late taking vitals at the right time because I’m just alone.* (IDI-002-Waithaka)

#### Sub-theme 1.3: limiting numbers

The participants from all the health facilities stated that they had to reduce the number of pregnant women and mothers seeking health services before and after delivery to reduce congestion during the early stages of COVID-19 as per social distancing rules. This led to some of the women missing health services. A health provider elaborated:

*Even here*,* it was not easy…. when they came*,* they were taken to the tent*,* and the social distance affected them. Then*,* they had a limited number of clients they could see daily. So*,* a mother can come even two days without being seen…* (IDI-012-Mutuini).

According to health providers, during the intense COVID-19 period, visitors accompanying the mother were not allowed in the health facility after admission as a measure of reducing overcrowding. Consequently, pregnant women lacked the support of relatives and friends during the delivery period. The health providers explained that relatives help mothers get warm water for bathing, bring home-cooked meals, and help with taking care of the baby after delivery– services not provided by the facility and its staff. A health provider said:

*One thing that happened during the COVID time was that visitors were not allowed totally. They would bring the mother after everything unless there is an emergency or a complication; that’s when the companion is called so that we can accompany the patient to the next facility. But there were no visitors*,* which means there was no food from home. But pre-Covid and post-Covid*,* those things are back (in place) that a mother can be visited by her relatives*,* and they can bring food any time of the day.* (IDI-001-Mutuini)

#### Sub-theme 1.4: limiting services offered

Some health providers said they were advised to minimize physical contact during the pandemic’s early stages to avoid contracting COVID-19. Therefore, some of the services that entailed physical examination were not provided. A health provider stated:

*When COVID was at its climax*,* we were not able to do a physical examination of the mother; we were not even able to listen to the heartbeat of the baby. So*,* we were just taking history…. We were not allowed to go any closer to the mother to examine that mother because of fear of infection…….* (IDI-007-Riruta)

From the interviews, health providers explained that mothers were allowed to stay in the hospital for 24 h pre-COVID-19 after giving birth. However, during the COVID-19 pandemic, mothers could only stay 6–8 h at the health facilities. According to the health providers, the limited time had an impact on some mothers who returned to the health facilities with complications that could have been avoided or dealt with before being discharged. Health providers explained:

*…… They did not stay here for long. The time was reduced; a mother could deliver and hold her for a few hours*,* less than 24 h*,* and maybe deliver in the morning and the evening they go. That was not good for the mother because maybe they went home*,* and the others developed some issues that could have been sorted out before they left to go home. You find them coming back*……(IDI-012-Mutuini).

*But if a delivery occurs at night they have to stay through the night till the following day. But if it is a day delivery*,* six to eight hours*…… (IDI-011-Waithaka).

Health providers also stated that weighing the children was suspended within the child welfare clinic, and the focus was immunization due to fear of exposing children to the COVID-19 virus. A health provider said:

*…. we never used to weigh babies or children coming for immunizations; those were the ones who were screened and allowed in…. we started weighing much later. I think we were also afraid of the pandemic; we felt that we would expose the babies to the pandemic if they came here.* (IDI-003-Waithaka)

Health providers said that some of the services, like family planning, were also limited, such as the long-term methods, because contact was required, and health providers feared contracting COVID-19. A health provider said:

*And also in the family planning*,* the services which were taking long*,* we couldn’t do them…(such as? ) Putting a coil….* (IDI-017-Mutuini)

#### Sub-theme 1.5: delayed service delivery

Health providers reported that due to the pandemic, they were required to follow some preventive measures such as donning personal protective gear, ensuring social distancing, and fumigation of the health facility if a pregnant woman or mother was suspected of having COVID-19. Health providers said they had to delay ANC and postnatal clinic appointments because of the preventive measures. A health provider stated:

*Like in postnatal*,* instead of telling them to come after two weeks*,* we only tell them to come if they experience any danger signs. The child should come after six weeks when they start their immunization to reduce the exposure for the child and the mother…… Other adjustments were at antenatal…. you just tell them to come after two months instead of one month.* (IDI-002-Waithaka)

### Theme 2: challenges experienced by health providers

Health providers reportedly experienced several challenges because of the COVID-19 pandemic and its prevention and infection control measures.

#### Sub-theme 2.1: fear of contracting COVID-19

Health providers said that in the early stages and intense period of COVID-19, they feared contracting COVID-19 because many people were succumbing to the virus, and there was limited information on the management of COVID-19. A health provider explained:

*Fear*,* trauma*,* anxiety. (Interviewer-What were you anxious about? ) You will be infected at any time. And the outcome of it*,* and how it will be. We ended up losing many colleagues*. (IDI-007-Waithaka)

#### Sub-theme 2.2: contracting COVID-19

Most of the health providers stated that they contracted COVID-19, with a severe impact on their physical and mental health. A health provider stated:

*I got very bad COVID*,* and then I was pregnant. I will never forget that I used to have difficulty breathing and chest pain. I thought I would die; at that time*,* I asked myself if I really wanted to practice or not…….* (IDI-016-Mutuini)

Some of the health providers who contracted COVID-19 at work and were taken to isolation centers were forced to pay for their health services and isolation costs. A health provider stated:

*When the health workers became sick*,* there was no waving or even paying for the hospital bills; there was no compensation….* (IDI-008-Waithaka)

#### Sub-theme 2.3: stigma

Most of the health providers reported experiencing stigma from their neighbors and community as they were associated with COVID-19, especially before COVID-19 vaccination was provided. Health providers explained:

*….Like where I was staying*,* my neighbors restricted their kids from playing with my kids because they told them that the doctor would come with corona to infect them. So there was that stigma.* (IDI-007-Riruta)

#### Sub-theme 2.4: overworking

Health providers also said that during COVID-19 peak times, they were overwhelmed with work due to the shortage of health providers. The shortage was created because some of the health providers were taken to work in the isolation centres. They said:

*Also*,* some of our colleagues were pulled from these facilities and taken to the COVID centers*,* which created shortages; we had to change duties from shifts to straight duties.….So the shifts became long…* (IDI-003-Waithaka).

The workload also increased with the introduction of the COVID-19 vaccines:

*At first*,* we were overwhelmed because when COVID-19 vaccination was introduced*,* our facility was among the first hospitals to offer vaccinations. Everybody from Dagoretti up to the neighbouring counties used to come to this facility for vaccination. So*,* it was very overwhelming.…. Again*,* with the vaccination*,* we were still doing the routine normal work…* (IDI-009-Mutuini).

#### Sub-theme 2.5: limited resources

One of the challenges the health providers reportedly experienced was a lack of PPEs. They stated that, at times, they had to use their own resources to purchase PPEs. They explained:

*Because we reached a point where we did not have any masks. That was the time COVID was a wildfire; everybody was so afraid*,* and then there were no masks…. some of us had to buy masks.* (IDI-003-Waithaka)

*Sometimes we could lack sanitizers*,* and we could buy for ourselves*,* we could buy masks*,* but they were so limited*,* and that’s how we could not reach a lot of households.* (IDI-004-Riruta)

Health providers also said that accessing ambulatory care and referral facilities was difficult. This was because there were few ambulances with a lot of demand from COVID-19 patients all over Nairobi County. The referral of patients to referral health facilities (higher-level facilities that are better equipped and capacitated to handle health emergencies) was challenging because they were full of COVID-19 patients. Health providers had stated that women who were pregnant but tested positive for COVID-19 were supposed to be referred to referral facilities that could assist such patients. However, ambulatory care for obstetric emergencies and pregnant COVID-19-positive women was inaccessible due to a high number of critical COVID-19 patients. A health provider explained:

*….the issue with an ambulance is you can have that patient (pregnant woman) brought in. You suspect this patient has COVID*,* and they know very well this patient is supposed to be evacuated*,* but you can call an ambulance forever…. So ambulance delays. Referral also because we used to refer to Mbagathi (hospital)*,* you would call and be told it is full*. (IDI-006-Riruta)

According to the health providers, in the initial months of the pandemic, the movement of persons and public transportation hours were limited due to curfew restrictions. Transportation services beyond 7 p.m. were limited. Consequently, movement and transport services were constrained, which affected health workers negatively, as elaborated below:

*….The public transport was not operating beyond 7. I’m here working in maternity*,* and someone who was supposed to release me hasn’t come*,* or even if someone has come*,* you have a case*,* and you must finish that case; you cannot leave a mother in the second stage who is delivering and hand over. You must complete and make sure the mother has delivered*,* and then when you go out there*,* there is no transport*. (IDI-012-Mutuini)

Some health providers reported that they lacked vehicles and relied on transport services from other facilities that were occupied during the COVID-19 pandemic. For some activities, such as picking up vaccines and conducting outreaches, they had to rely on erratic public transport, which was challenging.

*Another one is now the transport to collect the vaccines. You know the government is not providing the transport*,* so you arrange yourself how you collect the vaccines. You know*,* the vaccines are being collected from the depot and we are not supported…. Even now*,* if you have brought the vaccines here*,* then you want to go to an outreach somewhere*,* but there is no vehicle to do that.* (IDI-005-Waithaka)

#### Sub-theme 2.6: psychological stress

Some of the health providers stated that they experienced mental stress from contracting COVID-19 and the isolation from family. They also feared infecting their families with COVID-19, which they would have contracted from work. Health providers said:

*……. during that period when COVID-19 was everywhere*,* I happened to contract it*,* and I was psychologically disturbed because I couldn’t be allowed to go home because there was that fear I could spread COVID to my loved ones. Imagine staying alone for a period of a week or so because you are not allowed to go see your family.* (IDI-001-Riruta)

### Theme 3: government response to challenges experienced

According to the health providers, the Kenyan government at the county and sub-county level attempted to address challenges experienced by health providers by training them on COVID-19 prevention and infection control, supply of PPEs and vaccination, tax relief, and facilitation of movement. However, these were often only partially implemented and partially successful.

#### Sub-theme 3.1: training on COVID-19 prevention and infection control

The government trained several health providers on infection control and management to help health providers deal with the fear of contracting COVID-19. The training helped dispel their fears, enabling health providers to offer services without limiting the number of clients. A health provider stated:

*… We got teams from the county or sub-county coming to do training (on COVID-19) within the facilities…. I think their training helped us come out of the fear*,* and we stopped minimizing the number of women we saw. So*,* women started receiving care as they came without limiting the numbers.* (IDI-003-Waithaka)

#### Sub-theme 3.2: supply of PPEs and vaccination

After complaints from health providers, the government made efforts to supply PPEs as explained by the health providers:

*They gave us PPEs because you must help the mother give birth; you can’t stay one meter away*. (IDI-005-Mutuini)

*We were supplied with masks by the government*. (IDI-001-Riruta)

The PPEs were supplied; however, they did not meet the demand of health providers.

When COVID-19 vaccinations arrived in Kenya, the government prioritized health providers with vaccination since they were frontline workers. Many of the health providers in our study welcome this development, as stated in the excerpts below:

*I think that’s all. The vaccine also when it came*,* the priority was given to healthcare workers. So*,* at least with the vaccine*,* we started feeling a bit safe*…. (IDI-003- Mutuini)

#### Sub-theme 3.3: tax relief

The health providers said the government also gave tax relief of 5% for about 3 months in 2020. Kenya’s usual tax amount is 30% of the salary for certain salary categories. A health provider explained:

*What the government provided was the first three months; I don’t know whether it was May*,* June*,* or July*,* but they didn’t tax us PAYE (Pay as You Earn)…… I think it was the 25%…* (IDI-003-Riruta).

This provided some economic relief, as living costs were increasing due to pandemic restrictions.

#### Sub-theme 3.4: travel pass

According to the health providers, the government allowed them to use their work identification cards to move around during curfew time to facilitate their movement. They could show their work identification cards to the police to allow them to move at night. A health provider explained:

*…. the only thing that came up was curfew because you could be allowed to move around for free because of that permit for essential services.* (IDI-001-Riruta)

Despite having the travel pass, those who relied on public transport were still affected as options were limited and more expensive compared to non-pandemic periods.

### Theme 4: future response/pandemic preparedness

When asked how the government could better prepare for future pandemics, health providers proposed increasing awareness and training on pandemics, setting aside financial and human resources, building isolation centers, and providing psychosocial support and motivation to health providers.

#### Sub-theme 4.1: awareness creation and training of health providers

Health providers stated that they would like to be trained on pandemic preparedness in the future to alleviate fear and provide them with knowledge to deal with pandemics. They also requested to have continuous training. In addition, they wanted the community to be sensitized on pandemics to alleviate anxiety. Health providers said:

*Training of staff*,* sensitization of communities*,* and preparedness to alleviate anxiety. Because with COVID*,* even before it arrived*,* there was so much tension*,* there was so much anxiety…*.(IDI-001-Mutuini).

*Also*,* these training sessions are supposed to be ongoing…. There is that tendency to forget (what one was trained on). People are being introduced to the service who were not there during the training. So*,* training should be continuous for emergency preparedness….* (IDI-012-Mutuini)

#### Sub-theme 4.2: financial and human resources

For future pandemic preparedness, health providers advised the government to set aside funds to manage the pandemic by purchasing the needed medical equipment and supplies. In addition, adequate staffing was also recommended:

*They should keep some funds aside for things like adding ambulances and drugs. And now*,* in an emergency*,* they should set aside places they can use*,* like the ICU. During that time*,* we suffered; our patients died because of the lack of an ambulance and a facility where you could take them and a lack of staff.* (IDI-002-Riruta)

*Also*,* the healthcare workforce needs to be reinforced because*,* during that time*,* there was a lot of shortage*…. (IDI-003-Mutuini)

#### Sub-theme 4.3: isolation centers

Few of the health facilities had permanent isolation centers. The temporary ones were being used for other functions. The recommendation was that the government build isolation centers in each facility. A health provider stated:

*One is the isolation that were there during COVID time; they should be maintained. Currently*,* from what I have seen*,* they have been preoccupied with other things…. we don’t have any isolation. And they should build other isolation centers around all facilities.* (IDI-005-Waithaka)

#### Sub-theme 4.5: counselling

Some of the health providers had experienced mental stress, and none received any counselling. Consequently, they requested that in the future, psychosocial support should be provided to enable them to deal with the devastating effects of the pandemic. A health provider explained:

*…because counseling can help you understand that if you get COVID-19*,* it’s not the end because there were a lot of psychological traumas. If now another pandemic comes and I experienced COVID negatively*,* then I will have post-traumatic stress disorder.* (IDI-007-Waithaka)

#### Sub-theme 4.6: motivation for health providers

Health providers also stated that the government can motivate them in the future. Some health providers had to foot their medical bills when they got sick from contracting COVID-19 because of their interaction with patients. Therefore, health providers wanted the government to compensate them for risks incurred to enable them to work effectively. They said:

*…the government needs to motivate healthcare workers because they are frontline workers so they can work effectively*. (IDI-015-Mutuini)

*If the health workers have become sick because they are the ones who are in contact with their patients*,* the government knows… so that if there is any infection*,* at least that staff is going to be compensated.* (IDI-008-Waithaka)

## Discussion

COVID-19 infection control and prevention measures negatively affected maternal health services. According to our study findings, some health facilities were closed temporarily due to the pandemic limiting women’s access to maternal health services. Similar findings have been reported elsewhere [14]. To manage COVID-19 infections, some health providers had to be redeployed to COVID-19 isolation centers. This led to a shortage of health providers to provide maternal health services and compromised the quality of care. Several studies have reported similar findings where a shortage of health providers during COVID-19 negatively affected the quality of maternal health services [34–36]. Recruiting more health providers during pandemics is needed to ensure quality care. Recruitment methods that have worked before include fast-tracking medical students to join the workforce, canceling healthcare workers’ leave, and drawing on retired health providers to take on duties that put them at lower risk of infection [37–39].

COVID-19 prevention measures affected maternal health services negatively in other ways, as some maternal health services offered had to be limited due to restrictions. During the onset of COVID-19, health providers were advised to limit physical contact with patients. Research conducted in Kenya, Uganda, and Zambia on the impact of the COVID-19 pandemic and policy response on access to and utilization of reproductive, maternal, child, and adolescent health services reported limited contact between health providers and clients [40]. Limited contact led to avoidance of physical exams during antenatal care, post-partum mothers being released less than 24 h after delivery, and long-term family planning methods not offered to women. A study on the indirect impact of the COVID-19 pandemic on utilization and outcomes for reproductive, maternal, newborn, child, and adolescent health services in Kenya reported a reduction in long-term family planning methods [41]. Meeting women’s unmet need for varied contraceptive methods preserves reproductive, maternal, and child health as they can space pregnancies and achieve their fertility intentions, promoting healthier families and communities [42]. Governments must ensure that preventive measures do not compromise healthcare quality for pregnant women and mothers. Poor quality of maternal health care contributes to maternal morbidity and mortality.

To avoid congestion and prevent the spread of COVID-19, health providers were advised to limit the number of clients seen daily, restricting access to maternal health services. Consequently, the support of birth companions was restricted, according to our findings. Similar findings were reported in a study on the impact of COVID-19 on the provision of respectful maternity care from a global survey of health providers where health facilities restricted birth companions during child labor and birth [34]. Research has shown significant associations between low social support and the risk of depression, anxiety, and self-harm during pregnancy [43].

Our study indicates that some measures observed for infection prevention and disease control, such as social distancing, postponing appointments, and fumigating health facility rooms, delayed access to services pregnant women and mothers sought. These findings align with a study conducted on the impact of COVID-19 on the provision of respectful maternity care [34]. Delayed maternal health services may negatively affect maternal health, maternal health-seeking behavior, and future utilization of maternal health services by pregnant women and mothers [44].

Our study findings show that health providers experienced several challenges due to the COVID-19 pandemic. They included contracting the disease and subsequently having to meet the health care and isolation costs, psychological distress, and stigma from the community. A study on risk perceptions and preventive practices of COVID-19 among healthcare professionals in public hospitals in Addis Ababa, Ethiopia, reported that the majority of health providers were worried about contracting COVID-19 from their roles, as well as transmitting it to their families and they worried about their mental health [45]. Another study on the risk of healthcare worker burnout during the COVID-19 pandemic found that 20% asserted daily depression symptoms during the pandemic compared to 2% before the pandemic [42]. Health providers’ fears centered on the risk of infection due to lack of resources (33%) and community transmission (23%), economic insecurity (11%), and social stigma (11%) [42].

Other challenges included limited resources where health providers sometimes lacked protective personal equipment and had to purchase it themselves. A South African study reported that health providers lacked PPE, increasing their risk of COVID-19 infection [46]. In addition, due to movement restrictions, transportation costs increased, subjecting health providers to increased personal expenses. Similar findings are reported elsewhere [47]. Research shows that COVID-19 negatively impacted job satisfaction among health providers, with inadequate preparedness, stress, and burnout as the significant contributing factors [48].

Since most of the challenges were experienced at the onset of COVID-19, the Kenyan government responded to some. To reduce the fear of contracting COVID-19, the government trained some health providers on infection control and management, supplied PPEs, and prioritized health providers for vaccinations. These interventions were implemented by several governments in sub-Saharan Africa [49]. Tax relief was offered temporarily; such incentives should last until the pandemic threat is lowered to cushion health providers against unnecessary high costs from inflation and infection control and prevention measures.

Health providers provided recommendations on future pandemic preparedness. They included community awareness creation and sensitization and training of health providers on the pandemic to alleviate fear, manage pandemics, and promote prevention and infection control measures. It was also recommended that financial resources be set aside to facilitate the construction of isolation centers, purchase medical supplies and equipment, and adequate staffing to manage the pandemic. Similar recommendations have been made elsewhere [39, 50]. The financial resources can also incentivize health providers by providing a risk allowance and catering for their medical expenses [47]. Psychosocial support for health providers was also recommended. A study on the impact of COVID-19 on health providers in 13 African countries reported an increase of 2-20% in daily depressive symptoms [51]. Motivation and psychosocial support for health providers have been recommended in studies on the impact of COVID-19 on health providers in Africa [39, 52]. Motivational incentives such as risk allowances should target al.l health providers as all run a risk of infection, although higher risks are born by those working on COVID-19 patients. There could be differences in the amounts provided; however, the differences should be equitable to promote solidarity among health workers.

### Strengths and limitations of the study

The study provides a perspective of health providers on the effect of COVID-19 on maternal health services and health providers, as well as measures taken by the Kenyan government to address them in Kenya. We interviewed different cadres of health providers from different health facilities, providing an in-depth scope of the effect of COVID-19 on maternal health services. The views of interviewed health workers offer insights into challenges that health workers faced elsewhere in the country, in delivering maternal health services. However, the views of the interviewed health workers serving urban informal settlements in Nairobi are not representative of the whole country and cannot be generalized.

## Conclusion

From the perspective of health workers on the frontline of maternal health, who provide services to mothers in Nairobi’s informal settlements, the COVID-19 pandemic, with the resultant infection and prevention measures, negatively affecting the provision of maternal health services, compromising the quality of maternal healthcare. Health providers experienced several challenges, such as fear of and contracting COVID-19, limited resources, feeling overwhelmed, stigma, and psychological distress. The Kenyan government addressed some issues by providing PPEs, vaccines, and temporary tax relief. Although the Kenyan government attempted to address some of the challenges, more must be done to tackle psychological distress and provide proper health insurance for health providers. Hiring extra health providers should be prioritized in pandemics to avoid overwhelming a constrained health care system. Proper planning and budgeting for future pandemics is key. Planning for pandemics should cater to proper staffing, training, motivation of staff, health insurance, and psychosocial support. In addition, funds should be set aside for medical supplies and equipment, vaccine purchases, isolation centers, and other contingencies.

## Electronic supplementary material

Below is the link to the electronic supplementary material.

Supplementary Material 1

## Data Availability

The anonymized data can be provided upon request, which will be approved by all the authors. To request data and materials, contact Dr. Cynthia Khamala Wangamati at cynthiawangamati@gmail.com.

## Abbreviations

WHO: World Health Organisation
COVID-19: Coronavirus disease
MMR: Maternal Mortality Rate
SSA: sub-Sahara Africa
TBA: Traditional Birth Attendants
IDI: In-depth Interviews
