# Supplementary material for: Health providers’ perspectives on effects of the COVID-19 pandemic and anti-epidemic measures on maternal health services in Nairobi, Kenya: a qualitative study
Source: BMC Pregnancy Childbirth. 2025 Apr 11;25:430. doi: 10.1186/s12884-025-07500-8 (PMC11992752; doi:10.1186/s12884-025-07500-8)
Supplement: Supplementary file 1 — Supplementary Material 1 [file 12884_2025_7500_MOESM1_ESM.pdf]

Supplementary Table 1: Key informant interviews with health providers- Interview guide

Demographics

Name

Age

Educational Background

Profession

Working experience

Training on maternal health

1. What is your role in provision of maternal health services?
   -Did your role change or did you take up extra tasks with the onset of the covid-19 pandemic?
   -How did the extra tasks affect your ability to provide care?
2. What services does your facility provide to pregnant women and mothers?
   -Were these services affected by the onset of COVID-19? Elaborate
   -Was delivery of any of these services hampered? How?
   -Did you have to adjust the delivery/ provision of these services? (Virtual services)
3. How was maternal health seeking behaviour affected by the onset of covid-19?
   -What adjustments did you make to promote health services?
4. Were pregnant women and mothers forced to seek maternal health services elsewhere due to COVID-19 restrictions? If yes, explain
   - How did you address this situation?
   - Are there any challenges related to this?
5. How were birth outcomes impacted by the onset of COVID-19?
6. With onset of COVID-19, resources were pooled from other health services to address the situation. How were maternal health services resources affected?
   -Human and financial
7. How has the government addressed the challenges experienced by women seeking maternal health services during the ongoing COVID-19 pandemic?
8. How has the government addressed challenges experienced by health providers working on maternal health?
9. What challenges have you faced during this ongoing COVID-19 pandemic period in your line of work?
10. How can these challenges be addressed? At the government level, at the facility level
11. How can the government and health facilities be better prepared for future pandemics?
12. Do you have any else you want to add or questions?
